# Usability of Minimal Invasive Surgery for Elbow Dislocation with Coronoid Process Fracture: A Protocol Development Study

**DOI:** 10.3390/life14080954

**Published:** 2024-07-29

**Authors:** Chun-Cheng Lin, Ming-Fai Cheng, Chien-Shun Wang, Chao-Ching Chiang, Yu-Ping Su

**Affiliations:** 1 Division of Orthopaedic Trauma, Department of Orthopaedics and Traumatology, Taipei Veterans General Hospital, Taipei 112, Taiwan; 2 National Defense Medical Center, School of Medicine, Taipei 112, Taiwan; 3 Department of Surgery, National Yang Ming Chiao Tung University, Taipei 112, Taiwan

**Keywords:** minimal invasive surgery (MIS), coronoid process fracture, elbow terrible triad, posteromedial instability, trans-olecranon, trans-ulnar basal coronoid fracture–dislocation, posterior Monteggia fracture

## Abstract

Objective: The study aims to explain whether or not minimal invasive surgery (MIS) would be feasible in elbow fracture–dislocation with coronoid process fracture. Methods: At Taipei Veterans General Hospital, patients who had elbow dislocations with coronoid process fractures underwent a single surgeon’s MIS techniques which included the fluoroscopy-guided ulnar anteromedial (FGUAM) approach in the stage of reducing the coronoid process. When there is a proximal ulnar fracture, the posterior incision should be necessary, followed by the incision over the lateral or medial elbow for treating radial fractures or ligament injuries. Results: The Flow Diagram for approach recommendation was established on the basis of defining MIS as that which does not include cross-plane dissection. The importance of anterior rigid fixation for the coronoid process was also emphasized. Conclusions: MIS can be achieved by multiple limited surgical incisions. Although the posterior extensile approach is necessary in situations of ulnar metaphysis or ligament avulsion fracture, the FGUAM approach decreases the cross-plane dissection.

## 1. Introduction

### 1.1. Background

Surgical treatment for fracture–dislocation of the elbow with a concomitant coronoid fracture is a challenge. Clinically, at the authors’ institute, the most common types are terrible triad fractures and Mayo’s basal-coronoid trans-ulnar fracture–dislocations, whereas posteromedial instability is the rarest. In such cases, the injuries include coronoid process fracture, radial head fracture, proximal ulnar fracture, and also ligament injuries. The most critical issue is not the degree of comminution, nor the sequence or technique for fixing these fragments, but restoring the stability of the elbow joint with the least damage to the soft tissue, and the patient must be able to engage in passive range of motion (ROM) training immediately post-operatively.

Although conventional extensive approaches can be used well for these fractures, such as the lateral extensile approach [1], fragment-specific approach with reflecting olecranon [2], medial extensile approach [3], or posterior approach extended to lateral and medial sides [4], there exist soft tissue problems and risk of nerve traction. The biggest potential problem is likely to occur in the case of too many cross-plane dissections, such as when those from the posterior plane through the medial plane and then to the anterior plane are performed in order to expose the coronoid process, especially in a situation with unstable ulnar metaphysis.

**Figure 1:**
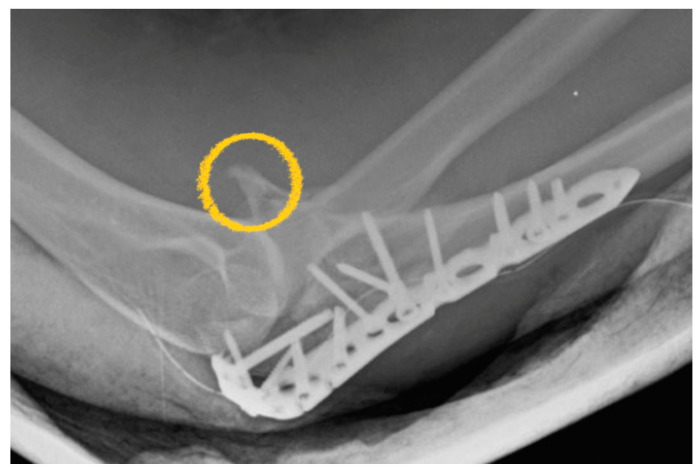
The coronoid process loss of reduction (yellow circle).

In the author’s experience, there was a case where the extended posterior approach was used but the coronoid process lost reduction (Figure 1). At that time, the coronoid process in this case could not be fixed with a screw from the rear side, so the Lasso technique was used to fix the fractured coronoid. However, during the immediate ROM training process after surgery, the avulsion force from the brachialis muscle was inevitable. Therefore, this made the author rethink the necessity of anterior fixation. In addition to the fixed effect, soft tissue dissection was also taken into consideration. The advantage of the front buttress plate is that it can directly provide better resistance to brachialis avulsion force.

In the authors’ institute, a kind of limited anterior approach is conducted by the experienced surgeon. This approach is named the fluoroscopy-guided ulnar anteromedial (FGUAM) approach and is supposed to be useful for reducing soft tissue damage in the treatment of elbow dislocation with concomitant coronoid fracture.

### 1.2. Objectives of the Study

Despite the fact that the best surgical method is still controversial, we believe that good results can be obtained with the treatment method that the surgeon is most familiar with. The authors aimed to establish a surgical protocol, the Flow Diagram based on the retrospective cases who had all undergone the FGUAM approach, explaining whether or not minimal invasive surgery (MIS) would be feasible in the situation of elbow dislocation with coronoid fracture. The focus of this study was to design a protocol composed of existing surgical techniques and experiences and, further, to show how to reduce soft tissue dissection. There are two categories in the Flow Diagram, MIS and non-MIS. Every step of the surgical technique is also discussed, including the FGUAM approach.

### 1.3. Hypothesis

The developed Flow Diagram based on the retrospective cases can facilitate the choice between MIS and non-MIS approaches, whereas the operator can find the clues for a treatment strategy in such complicated fracture types.

## 2. Materials and Methods

Three groups of cases were identified in the study. One had anteromedial facet fractures of the coronoid process in a situation of posteromedial instability, and another one had transverse coronoid process fractures in terrible triad fracture–dislocations. The third group had Mayo’s basal-coronoid trans-ulnar fracture–dislocations [5]. One single surgeon treated all cases with the same surgical principles. At the authors’ institution, these kinds of patients account for only a small proportion of all fractures, and only a few surgeons use the FGUAM approach. Therefore, the extent of the fracture types covered by the included patients was more essential than the number of cases when developing a protocol from the existing surgical techniques.

### 2.1. Pre-Operative Evaluation and Defining Minimal Invasive Surgery

A CT scan with three-dimensional (3D) reconstruction is supposed to be an essential survey for pre-operative planning. The 3D CT scan is used to identify tiny bone fragments such as the comminuted coronoid base, the avulsed fragments, and the shattered radial head. It is easy to check every fractured fragment in 3D images, especially the avulsion part of ligament insertion. The surgical approach window is quite different depending on whether the ligament is severed in the middle or inserted. The definition of MIS for the elbow was considered by the authors as it does not only depend on the absolute length of the wound, but also on whether the dissection spans different surgical planes. Furthermore, MIS of the elbow is defined as fractures of four planes treated by approaches on their respective planes without cross-plane dissection, and all are limited approaches or MIPPO. On the other hand, the cross-plane approach was considered as non-MIS. As for the surgical planes, different planes indicate the need for fixing different structures. For example, the buttressing coronoid process needs the anterior plane, whereas the avulsed ligament insertion or fractured proximal ulnar bone should be treated through the posterior plane.

In summary, the inclusion criteria for MIS in this study are for confirming whether the surgical wound on each plane during the operation is a limited approach, and there is no cross-plane dissection. In addition, the reduction and fixation sequence are set to four steps. The surgical techniques for each step are described below.

### 2.2. Steps of Fixing Coronoid Process Anteriorly

According to the above definition of MIS, if the surgeon expects to avoid cross-plane dissection, anterior fixation for the coronoid process is inevitable.

**Figure 2:**
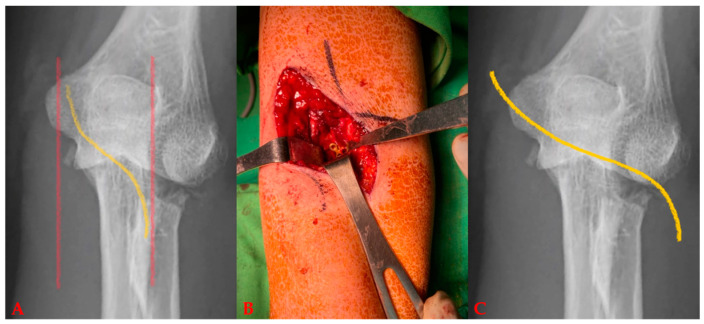
The fluoroscopy-guided ulnar anteromedial (FGUAM) approach: (**A**) under a plain radiograph, the curvilinear incision (yellow line) starts from the top of the ulnar border (red line) of the olecranon process, extending distally to the top of the bicipital tuberosity, (**B**) anterior fixation with a plate, (**C**) the original AM approach has the S-shaped incision (yellow line) from the ulnar side to the radial side in the antecubital fossa.

**Figure 3:**
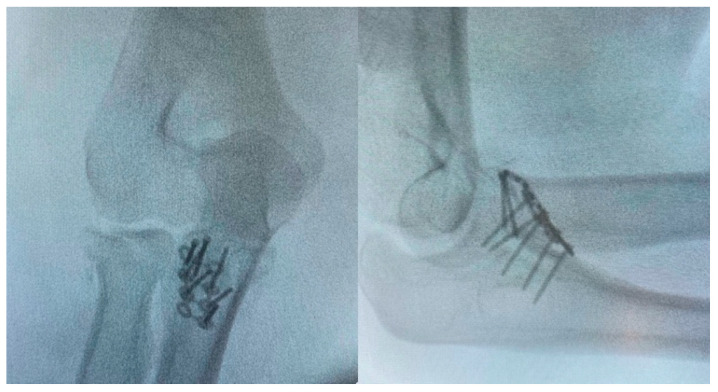
Fixation of the coronoid process with interfragmentary headless screws and a buttress plate.

**Figure 4:**
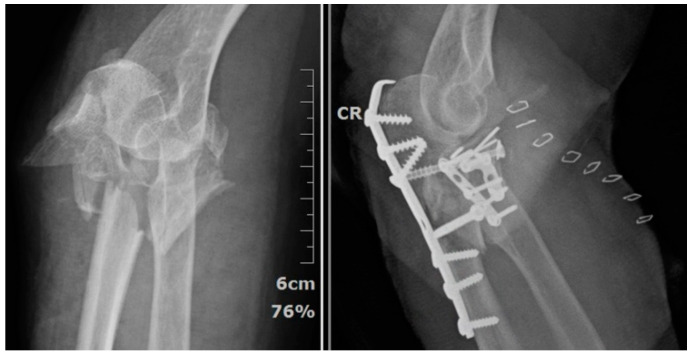
The unstable coronoid base should be restored before the fixation of the coronoid process.

There are 2 steps in the surgical techniques when fixating the coronoid process anteriorly. In the first step, the fractured coronoid process is exposed with the FGUAM approach (Figure 2A,B), which was modified from the original AM approach (Figure 2C) introduced by Yang et al. [6]. The original AM approach can be minimized by the FGUAM approach. When reducing the coronoid fragment, one 1.25 mm K-wire is perpendicularly applied. Then, the anatomical position of ulnohumeral joint is confirmed under the “splinting force” of the K-wire. A 1.5 mm headless compression screw can be chosen and applied just near to the splinting K-wire. In the second step, another buttress plate is used on the coronoid slope (Figure 3). Finally, the provisional K-wire can be exchanged for another 1.5 mm headless compression screw. However, if the base of the coronoid process is broken, the keystone step is not to fix the coronoid process but to restore the ulnar metaphysis first (Figure 4).

### 2.3. Fixation of Radial Head

**Figure 5:**
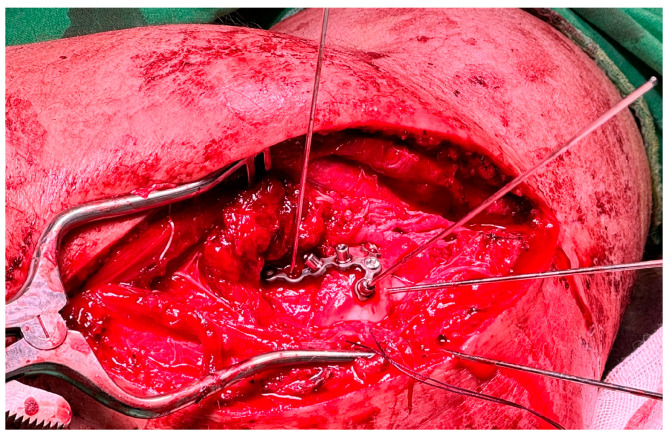
The hand system plate being applied to the radial head fracture.

According to the authors’ principle, radius fixation is performed only after the fixation of the coronoid process. The fracture site is exposed through the Kocher interval [7] and undergoes screw and/or plate fixation. In the author’s hospital, a hand system plate is used as the main fixator for radial head fracture (Figure 5). Especially for Asian females with smaller bones, the specific plate for the radial head is often too bulky to pass through the safe zone [8], causing impingement.

### 2.4. Variant Proximal Ulnar Fractures and Ligaments Injuries

**Figure 6:**
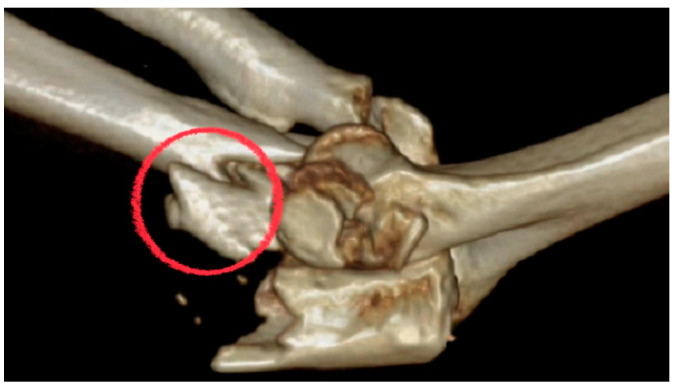
The base of the lateral ulnar collateral ligament is avulsed (red circle).

**Figure 7:**
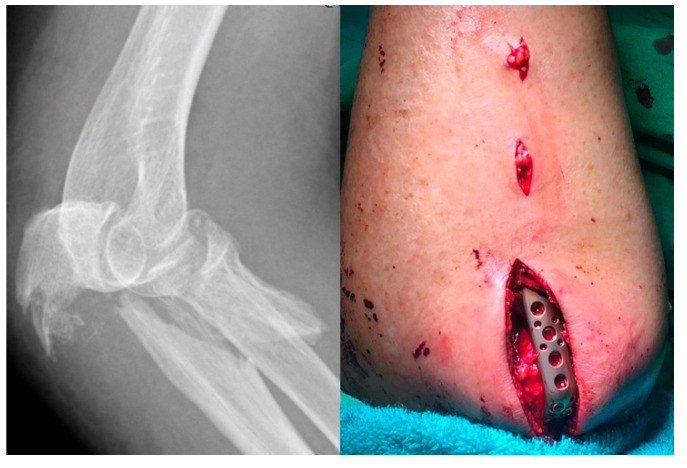
The relatively stable structure of the proximal ulna can be fixed with the posterior minimally invasive percutaneous plate osteosynthesis (MIPPO) approach.

When analyzing comminuted proximal ulnar fractures and surgical approaches, the authors did not rely on the classification of variant trans-olecranon fracture–dislocation [9] or Monteggia-like fracture–dislocation [10]. We chose the Mayo classification [5], which mentions the fracture as a basal-coronoid trans-ulnar fracture, and preferred to classify the approach methods into 2 types depending on ligamentous condition rather than the fracture type itself. If the preoperative assessment shows avulsion in the ligament insertion (Figure 6) or the ulnar metaphysis is very unstable, then the extensile posterior approach must be used to repair the ligament. If the ligament insertion base is complete and the ulnar metaphysis is relatively stable, then the posterior minimally invasive percutaneous plate osteosynthesis (MIPPO) approach could fix the proximal ulnar fracture (Figure 7). Finally, the elbow stability is checked again under C-arm fluoroscopy to determining whether or not the collateral ligament should be repaired through the lateral or medial surgical window.

### 2.5. Post-Operative Rehabilitation

The protocol for using a post-operatively functional brace is presented as a Gantt Chart in the study (Table 1). Under the protection of a functional brace, thirty degrees of passive ROM training can be achieved immediately within the first month after surgery. Then, it increases by 30 degrees every month.

## 3. Results

During the case collection process, it was found that patients who did not receive the anterior approach to fix the coronoid process sustained more soft tissue dissection during the operation. Eight cases with limited approaches were included in this protocol development. There were three cases of terrible triad fractures, only one case of posteromedial instability, and four cases of basal-coronoid trans-ulnar fracture–dislocations. All patients had a functional arc of the elbow and full forearm rotation not later than 3 months after the operation. There was no malunion or heterotrophic ossification.

**Figure 8:**
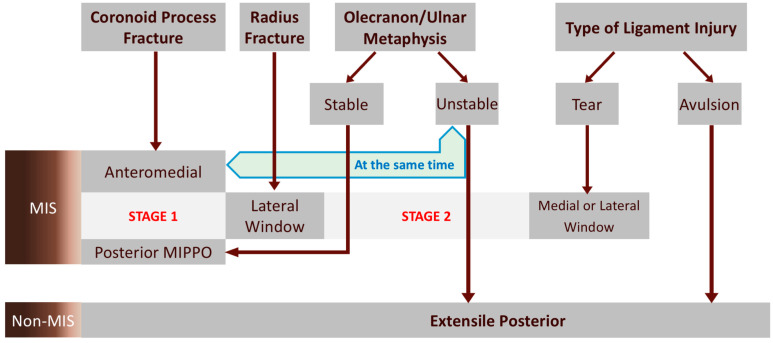
The Flow Diagram for approach recommendation was established on the basis of defining minimal invasive surgery (MIS) as that which does not include cross-plane dissection. The sequence of the upmost boxes from the left side to the right side represents the recommended sequence for reduction and fixation. The ulnar metaphysis and coronoid process must be fixed at the same time when the coronoid process base is unstable.

These comprehensive fracture types were enough to form a unique Flow Diagram. There are two main stages and two interval stages in this Flow Diagram developed by the authors. The Flow Diagram is shown in Figure 8. The four general steps of the fixation sequence are just shown on the top boxes, from the coronoid process to ligament injury. “Stage 1” is the first survey to evaluate whether or not coronoid process fixation could be the first-step surgery. If the coronoid fracture should be fixed, the AM approach with the FGUAM method is recommended by the authors. “Stage 2” is the second survey to check the condition of the proximal ulna. The two interval stages are lateral and medial windows for treating radius fracture and ligament injury, respectively. The lateral window is for treating the radial head as well as for the lateral collateral ligament. The lateral collateral ligament can only be treated through the lateral window if it is not an insertion avulsion fracture. In detail, the insertion site anatomically belongs to the lateral ulnar collateral ligament, and we surgeons need the posterior approach through the Boyd interval to expose it. Finally, in the Flow Diagram, the medial window in the last step is for treating the medial collateral ligament.

However, in the case of basal-coronoid tran-ulnar fracture–dislocation, it is often impossible to fix the coronoid process independently first, and the ulnar metaphysis and coronoid process must be fixed at the same time. In this kind of situation, the comminuted proximal ulna bone is reduced through two surgical windows, the AM and posterior incisions, synergistically. Therefore, AM and posterior MIPPO approaches can be carried out in the same stage: Stage 1. In the group of MIS, all approaches are performed with limited incisions.

As shown in the Flow Diagram, if there is an unstable ulnar metaphysis or ligament avulsion fracture, the posterior incision has to be extended to the medial or lateral plane alongside the ulnar bone. In such a case with an extended posterior approach, the so-called MIS fails to be established. Even if the extensile posterior approach is used in the case of unstable metaphysis and avulsion fracture of ligaments, it is still recommended to use the FGUAM approach for coronoid process fracture.

In theory, up to four separate approaches can appear according to this Flow Diagram, but there is no case with more than three approaches. In reality, if the fracture–dislocation is complex enough to require four approaches, it will be accompanied by unstable metaphysis or avulsion fracture of ligaments. Therefore, the extensile posterior approach will extend to cover the lateral or medial incision.

## 4. Discussion

The authors have tried to explain the proof of concept about MIS approaches but not the outcome analysis of the protocol application. The study Results are the protocol itself, not the clinical outcomes of the included cases.

### 4.1. Novel Achievements of the Study

The authors provided a specific protocol to illustrate the reduction sequence and the conditions under which MIS for elbow fracture–dislocation can be achieved, and the conditions required for non-MIS to be used can be known during preoperative planning. These were not explicitly mentioned in the previous literature. The protocol emphasizes the importance of anterior fixation for the coronoid process. In the Flow Diagram, regardless of whether MIS is available, it is recommended to use the FGUAM approach when the coronoid process fracture should be fixed because this approach can avoid cross-plane dissection and increase the feasibility of placing a buttress plate from the anterior side.

### 4.2. Classification of Comminuted Proximal Ulnar Fractures

In the past, there was a bit of confusion in the classification of proximal ulnar fracture combined with coronoid process fracture, especially when surgeons had to distinguish between Monteggia-like fracture–dislocation and variant types of trans-olecranon fracture–dislocation. Haller et al. [11] pointed out that the difference between a Monteggia-like fracture and a trans-olecranon fracture lies in whether the proximal radioulnar joint (PRUJ) is dislocated. In fact, it is not easy to confirm whether the PRUJ is truly connected when the coronoid base is separated from the ulnar metaphysis. Therefore, we recommend the Mayo coronoid-centric classification system published by O’Driscoll et al. [5], which can provide more concise communication and planning before surgery. In the case of trans-ulnar fracture, only when the coronoid process fracture needs to be fixed will the so-called cross-plane dissection be considered. In other words, it is whether or not an extensive approach from the posterior (or lateral or medial) to the anterior plane is necessary to fix the coronoid process.

### 4.3. Approaches for Elbow Dislocation with Coronoid Fracture in the Literature

The authors consider that the approaches to elbow dislocation with coronoid fracture should be divided into two categories for discussion. In one group, the coronoid process is fractured but the coronoid base is intact, which is commonly seen in posterior medial instability and terrible triad fractures. Huh et al. compared the exposure through the medial elbow using the flexor carpi ulnaris (FCU)-Splitting and Hotchkiss Over-the-Top approaches [12]. Hou et al. reported the single lateral approach for terrible triad injuries [1]. Zeiders et al. proposed a posterior global incision to restore complicated outcomes of unstable elbow joints [13]. In addition to the single surgical approach of medial, lateral, or posterior incision, some studies also suggest a two-incision technique [14,15].

In another group, there is the unstable coronoid base, whether it is called a type IID posterior Monteggia fracture [16], trans-olecranon fracture, or basal-coronoid trans-ulnar fracture. Authors of the earlier literature, years ago, proposed a fragment-specific approach that they believed could be used for most of these kinds of comminuted fractures [2]. There is also the posterior incision extended to the lateral side with a modified Boyd approach presented by a recent study in which the osteotomy of supinator tuberosity is performed [17]. However, the anterior buttress plate is still difficult to accomplish with this method.

Compared with other structures, the coronoid process is located alone in the anterior plane, which means that, if a single surgical approach is to be used, a larger soft tissue dissection will be required to reach the cross-plane, especially when rigid anterior fixation is necessary. Therefore, relying on a single surgical approach often damages more soft tissues.

### 4.4. Challenges in the Flow Diagram

Among these protocols, the most challenging one is the modified AM approach: the fluoroscopy-guided ulnar AM approach. Whether it is treating posteromedial instability, terrible triad fracture, or basal-coronoid trans-ulnar fracture–dislocation, the authors consider that the rigid anterior fixation is essential and recommend using the FGUAM approach to decrease soft tissue damage during the fixation of the coronoid process. Compared with the original AM approach, the FGUAM approach requires fluoroscopic assistance to localize the range of the skin incision according to the shape and position of the proximal ulna under a plain radiograph. The curvilinear incision starts from the top of the ulnar border of the olecranon process, extending distally to the top of the bicipital tuberosity (Figure 2A). After incision of bicipital aponeurosis and dissection through the interval between the brachial artery and median nerve, it is often easy to find the fractured fragment of coronoid process just in the brachialis muscle. As for the next reduction and fixation, they have a learning curve and also some pitfalls. The critical landmark is the bicipital tuberosity where the brachial artery and median nerve begin to branch. Thus, the dissection and plating are recommended to not exceed that landmark. Because of the limitation of this landmark, the anterior buttress plating can only fix the coronoid process. However, the unstable comminuted coronoid base cannot be well fixed by the anterior plate alone, and additional screw fixation from the posterior side is necessary.

### 4.5. Implants Selection

According to the author’s experience, the size of the interfragmentary screw used to fix the coronoid process is more suitable when it does not exceed 2.0 mm. However, the screw size of the anatomic locking compression plate (LCP) exclusively for the coronoid process currently on the market is generally larger than 2.0 mm. Thus, the authors recommend using a hand system LCP and the buttress plate directly in front as the main fixation method. The same issue will also occur in radial head fracture. In Asian females with smaller bones, the hand system LCP can better stay in the safe zone of the radial head.

### 4.6. Limitations of the Study

As it is a proof of concept, there was no control group in the study. The developed protocol is practical, but there was no comparison to other methods. Although the fracture types were comprehensive, there was also no consensus about how many cases are needed to develop a surgical protocol. In spite of developing the protocol for surgical techniques exclusively for the MIS concept, the good outcome was not the hypothesis of the study and was not guaranteed by the protocol either.

### 4.7. Future Applications of the Research

In the literature, the treatment methods for elbow fracture dislocation with coronoid process fracture are mainly based on the experts’ specific techniques, and there is no protocol that considers soft tissue dissection or whether MIS can be performed. By promoting the protocol proposed by the authors in the study, we expect that surgeons pay attention to two key points: how to reduce soft tissue dissection through the anterior approach and the stability condition around the ligament insertion. If there is no instability near the ligament’s insertion, the extensile approach is probably not necessary. Based on the proof of MIS concept in the study, a further randomized controlled study in the future probably can be conducted to analyze treatment outcomes.

## 5. Conclusions

Despite lacking prospective clinical application and analysis of the protocol, the study is a proof of concept that MIS can be achieved by multiple limited surgical incisions. The key point in the protocol is that reducing and fixing the coronoid process back to the proximal ulna needs the FGUAM approach with more reliable anterior fixation and does not rely on cross-plane dissection, especially in situations of unstable metaphysis or ligament avulsion fracture. From another perspective, if the bone structure near the ligament’s insertion is stable, the posterior extensile approach for proximal ulnar fracture can be replaced by posterior MIPPO.

## Figures and Tables

**Table 1:** The schedule of post-operatively functional brace.

Week after OP	1	2	3	4	5	6	7	8	9	10	11	12
60–90 Degrees												
45–105 Degrees												
30–120 Degrees												
Follow-up X-ray												

## Data Availability

The data presented in this study are available on request from the corresponding author.
